# Tracheal injury detected immediately after median sternotomy by inexperienced surgeons: two case reports

**DOI:** 10.1186/s13256-018-1591-5

**Published:** 2018-02-27

**Authors:** Jun Takeshita, Kei Nishiyama, Atsushi Fukumoto, Suguru Ohira, Satoru Beppu, Nozomu Sasahashi, Nobuaki Shime

**Affiliations:** 1 Department of Intensive Care Medicine, Osaka Prefectural Hospital Organization, Osaka Women’s and Children’s Hospital, 840 Murodo-cho, Izumi, Osaka, 594-1101 Japan; 2 Department of Emergency and Critical Care Medicine, National Hospital Organization, Kyoto Medical Center, 1-1 Fukakusa, Mukaihata-cho, Fushimi-ku, Kyoto, 612-8555 Japan; 3 Department of Cardiovascular Surgery, Rakuwakai Otowa Hospital, 2 Otowachinji-cho, Yamashina-ku, Kyoto, 607-8062 Japan; 4 Division of Cardiothoracic Surgery, Department of Surgery, Thomas Jefferson University Hospital, 1025 Walnut Street, Philadelphia, PA 19107 USA; 5 Department of Emergency and Critical Care Medicine, Institute of Biomedical & Health Sciences, Hiroshima University, 1-2-3 Kasumi, Minami-ku, Hiroshima, 734-8551 Japan

**Keywords:** Trachea, Sternotomy, Electrocoagulation, Iatrogenic disease

## Abstract

**Background:**

Although median sternotomy is standard during cardiac surgery, the procedure is associated with a risk of injury to mediastinal organs. Here, we discuss two cases of tracheal injury following median sternotomy during cardiac surgery.

**Case presentation:**

Ventilation failure occurred in a 78-year-old Japanese man and a 71-year-old Japanese man after median sternotomy, and tracheal injury was identified. The sites of injury were directly repaired and covered with mediastinal fat tissue, following which ventilation was successful. The burn-like deposits observed at the site of tracheal injury and on the removed endotracheal tube support the notion that the injuries in our patients were caused by electrocautery prior to median sternotomy. In one case, short sternotracheal distance may have contributed to tracheal injury during post-sternal manipulation. In both cases, the relative inexperience of both surgeons also supports the suspected cause of injury.

**Conclusions:**

Tracheal injury represents a potential complication following median sternotomy, especially when performed by inexperienced surgeons or in cases of short sternotracheal distance. Anesthesiologists should consider this rare yet potentially lethal complication.

## Background

Although median sternotomy is standard for many types of cardiac surgery, the procedure is associated with a risk of injury to mediastinal organs. However, few reports have discussed tracheal or endotracheal tube injury caused by median sternotomy [1–5]. Among these, one case was caused by the electrocautery procedure prior to median sternotomy [5]. Here, we discuss two cases of tracheal injury potentially caused by electrocautery before median sternotomy by inexperienced surgeons. Informed consent was obtained from the patients and their families to report the details of these cases.

## Case presentation

### Case 1

A 78-year-old Japanese man with angina pectoris was scheduled for elective off-pump coronary artery bypass grafting under general anesthesia. General anesthesia was induced using fentanyl (0.15 mg), midazolam (5 mg), ketamine (30 mg), and rocuronium (70 mg). An endotracheal tube (Lo-Contour Oral/Nasal Tracheal Tube Cuffed Murphy Eye; COVIDIEN, Dublin, Ireland; internal diameter 8.0 mm) was smoothly inserted, and no ventilation abnormalities were observed. A pulmonary artery catheter was placed in his right internal jugular vein, and a transesophageal echocardiography probe was inserted without complications. General anesthesia was maintained using oxygen (1 L/minute), air (5 L/minute), and sevoflurane (1.5%).

**Fig. 1:**
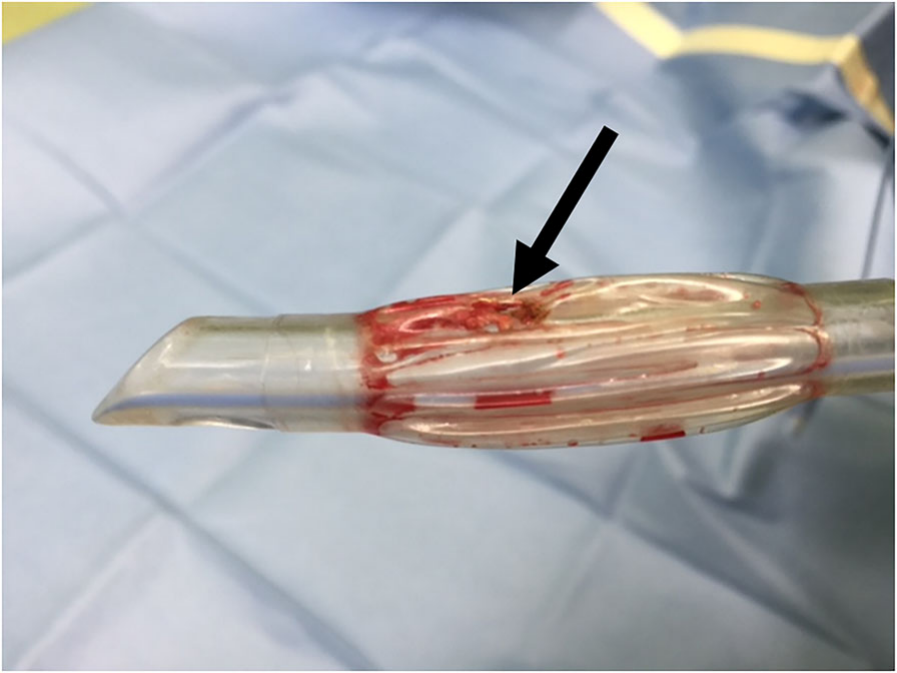
The cuff of the removed endotracheal tube had ruptured and exhibited burn-like deposits in case 1 (*arrow*)

Median sternotomy was performed by a resident physician with 1 year of experience in cardiac surgery. A caudal-to-cephalad incision was made using a sternum saw (Stryker Sternum System 7; Stryker Instruments, Kalamazoo, Michigan, USA). The anesthesiologist deflated the lungs during the sternotomy. Immediately after the sternotomy, the ventilator (Fabius GS; Drägerwerk AG & Co. KGaA, Lübeck, Germany) alarm sounded, indicating low tidal volume and minute volume. As ventilation was volume controlled, tidal volume could not be measured. Manual ventilation was also unsuccessful. It was confirmed that the ventilator circuit was connected appropriately. Sounds indicative of air leak were heard from the operating field, following which the surgical team identified an approximately 5 mm area of tracheal injury. Following re-intubation, a new tube was placed just above the carina, so that the cuff was located below the injury site under bronchofiberoptic guidance in order to avoid cuff injury and gain proper ventilation during reparation of the trachea. The tidal volume was recovered to normal, and vital signs, including blood oxygen saturation (SpO_2_), were normal and unchanged during reparation after re-intubation. We observed that the cuff of the removed endotracheal tube had ruptured and contained burn-like deposits (Fig. 1). Tracheal injury was directly repaired using absorbable sutures (PDS Plus 3–0; Johnson & Johnson K.K., Tokyo, Japan), and covered with mediastinal fat tissue. It required approximately 20 minutes to repair the trachea from the time we noticed the injury. After the injury had been repaired, ventilation was successful, and the operation proceeded uneventfully. Our patient was discharged from the hospital on postoperative day 28.

### Case 2

**Fig. 2:**
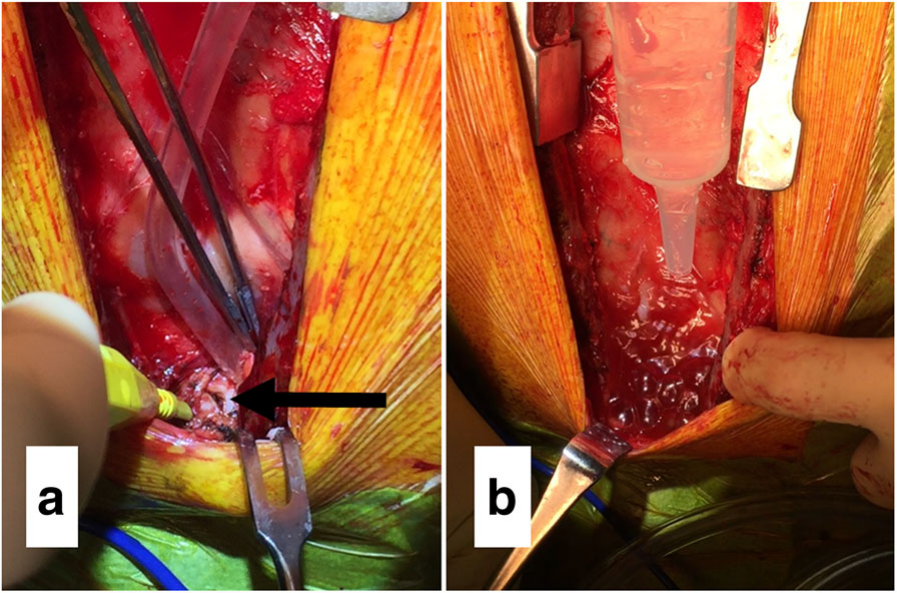
**a** Site of tracheal injury in the operating field in case 2 (*arrow*). **b** Air leak from the site of injury during lavage with normal saline in case 2

**Fig. 3:**
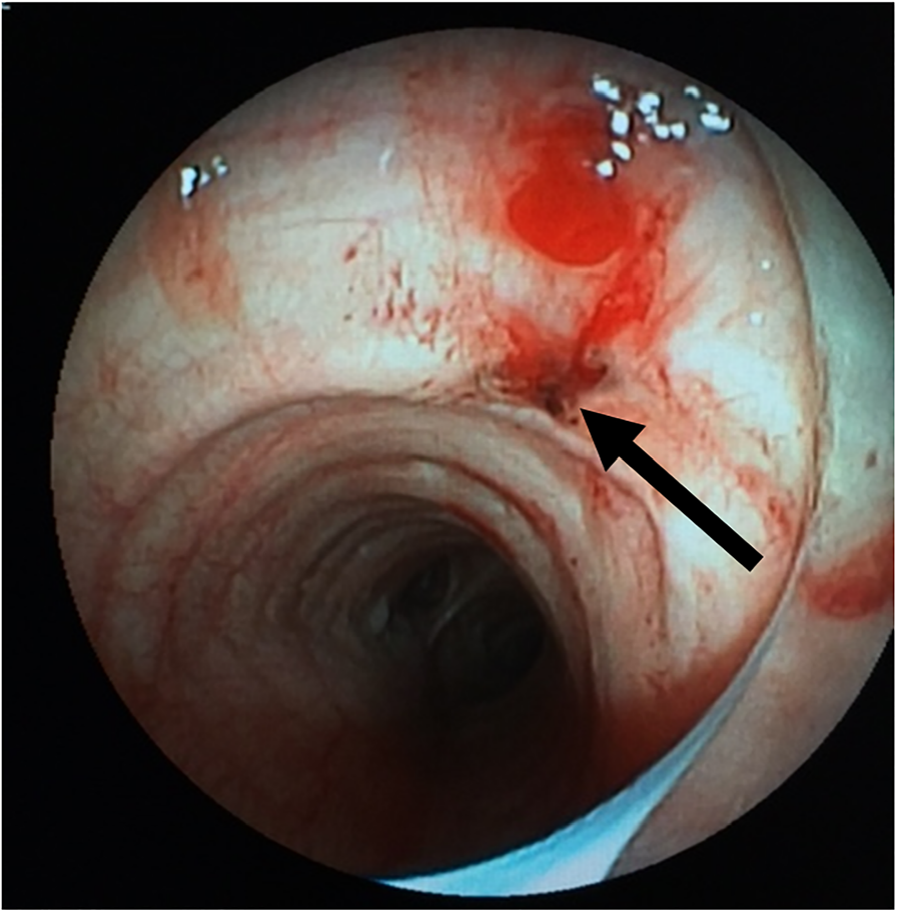
Tracheal injury accompanied by burn-like deposits was observed just distal to the tip of the tracheal tube under bronchofiberoptic observation in case 2 (*arrow*)

A 71-year-old Japanese man with angina pectoris was scheduled for elective off-pump coronary artery bypass grafting under general anesthesia. Preoperative preparations, including the induction of general anesthesia, were identical to those described for case 1. An endotracheal tube (Lo-Contour Oral/Nasal Tracheal Tube Cuffed Murphy Eye; COVIDIEN; internal diameter 8.0 mm) was smoothly inserted, and the initiation of positive-pressure ventilation was uneventful. Median sternotomy was performed using a sternum saw by a cardiovascular surgeon who had performed fewer than 150 such procedures. The anesthesiologist deflated the lungs during the sternotomy. Immediately following median sternotomy, tidal volume had decreased from 500 mL to approximately 200 mL under volume-controlled ventilation. It was confirmed that the respiratory circuit was appropriately connected. Sounds indicative of air leak were heard from the operating field, following which the surgical team identified an approximately 3 mm tracheal injury (Fig. 2a, b). A bronchofiberoptic examination revealed that tracheal injury had occurred just distal to the tip of the tracheal tube, which exhibited burn-like deposits (Fig. 3). The anesthesiologist confirmed that the cuff of the tracheal tube was intact. The tracheal tube was advanced and placed just above the carina, so that the cuff was located below the injury site under bronchofiberoptic guidance in order to avoid cuff injury and gain proper ventilation during reparation of the trachea. The tidal volume was recovered to normal, and vital signs, including SpO_2_, were normal and unchanged during reparation. The site of injury was directly repaired using absorbable sutures (PDS Plus 3–0; Johnson & Johnson) and covered with mediastinal fat tissue. It took approximately 20 minutes to repair the trachea from the time we noticed the injury. Following repair of the injury, no air leaks were observed, and the perioperative course was uneventful. Our patient was discharged from the hospital on postoperative day 23.

## Discussion and conclusions

Although iatrogenic tracheal injury may occur due to tracheal intubation and surgical procedures, such complications are rare [6–9]. Only five case reports to date have discussed tracheal injury caused by median sternotomy during cardiac operations [1–5]. Among these, one case was caused by the electrocautery procedure used to detach the tissue under the upper end of the sternum prior to median sternotomy [5]. Similarly, the burn-like deposits observed at the site of tracheal injury and the cuff of the removed endotracheal tube support the notion that injuries in our patients were also caused by electrocautery prior to median sternotomy. The relative inexperience of both surgeons also supports the suspected cause of injury.

The distance from the posterior surface of the sternum to the anterior surface of the trachea (that is, sternotracheal distance), measured using preoperative computed tomography, was 24.5 mm in case 1 and 11.8 mm in case 2. Given that the distance has been reported as 17.4 mm and 19.2 mm with and without anastomotic leakage following esophagectomy [5], respectively, the short distance observed in case 2 may have contributed to tracheal injury during post-sternal manipulation.

Our findings indicate that tracheal injury represents a potential complication following median sternotomy. Inexperienced surgeons and supervisors should be very cautious regarding this complication especially in cases of short sternotracheal distance, as this complication is unacceptable. In case of sudden ventilation failure occurring before or after median sternotomy, anesthesiologists should be aware of this rare yet potentially lethal complication. Careful monitoring of ventilatory parameters during the peri-sternotomy period is recommended.
